# A do it yourself (DIY) point-of-care wrist ultrasound phantom for joint access training

**DOI:** 10.1186/s13089-024-00374-5

**Published:** 2024-06-14

**Authors:** Andrea Cheng, Justin Zhou, Chun Hei Ryan Chan, Connie Chen, Charlotte Cheng, Kaitlyn Storm, Anson Zhou, Alan Mao, Won Jun Kuk, Tiffany C. Fong, Ignacio Villagran, Constanza Miranda

**Affiliations:** 1 Department of Biomedical Engineering, Johns Hopkins University, Baltimore, MD USA; 2 Department of Emergency Medicine, Johns Hopkins School of Medicine, Baltimore, MD USA; 3 Department of Health Sciences, Faculty of Medicine, Pontificia Universidad Católica de Chile, Santiago, Chile

**Keywords:** Point of care, DIY, Task trainer, Arthrocentesis

## Abstract

**Background:**

Joint access is essential for arthrocentesis, or joint aspiration of fluids. Joint treatments that are not performed properly can result in avoidable patient issues such as damage to the muscles, tendons, and blood vessels surrounding the joint. The use of ultrasound has become the gold standard for this procedure and proven to be a support in the skill learning process. However, success with this equipment, particularly in small joints like the wrist, depends on a clinician's capacity to recognize the crucial landmarks that guide these procedures. Prior to executing on a real patient, task trainers have proven to be an effective way for doctors to practice and prepare for procedures. However, shortcomings of current solutions include high purchase costs, incompatibility with ultrasound imaging, and low reusability. In addition, since this is a procedure that is not performed frequently, there may not be space or resources available in healthcare facilities to accommodate one at the point of care. This study aimed to close the existing gap by developing a DIY ultrasound compatible task trainer for wrist joint access training.

**Results:**

We developed a novel ultrasound compatible wrist joint model that can be made from sustainable materials and reusable parts, thus reducing the costs for acquisition and environmental impact. Our model, which was produced utilizing small-batch production methods, is made up of 3D-printed bones enclosed in an ultrasound-compatible gelatin mixture. It can be easily remade after each practice session, removing needle tracks that are visible under ultrasound for conventional phantoms. The ultrasonic properties of this model were tested through pixel brightness analysis and visual inspection of simulated anatomical structures.

**Conclusion:**

Our results report the advantages and limitations of the proposed model regarding production, practice, and ultrasound compatibility. While future work entails the transfer to patients of the same skill, this reusable and replicable model has proven, when presented to experts, to be successful in representing the physical characteristics and ultrasound profile of significant anatomical structures. This novel DIY product could be an effective alternative to teach procedures in the context of resource-restrained clinical simulation centers.

**Supplementary Information:**

The online version contains supplementary material available at 10.1186/s13089-024-00374-5.

## Background

Arthrocentesis is a procedure done to obtain synovial fluid from a joint capsule for diagnostic or therapeutic purposes [1–4]. Failure to access the joint space may have drastic consequences on the efficacy of joint aspiration and injection [5]. In the emergency room, arthrocentesis is primarily used for diagnostic purposes. Through collection of synovial fluid, clinicians can diagnose inflammatory diseases that may require chronic management (i.e., arthritis, gout) or conditions like septic arthritis (joint sepsis) that may require acute intervention [6]. If arthrocentesis is not performed properly or in a timely manner, unidentified, and hence untreated, cases of joint sepsis may result in serious complications including loss of bone density, chronic pain, bone infection, and in the worst case, death. Therefore, the competence of the practitioner in performing this procedure and how the skill is acquired becomes especially relevant.

Arthrocentesis is typically not taught during medical school training, and many students perform this procedure for the first time on a live patient during residency. This lack of experience could lead to multiple attempts in order to successfully access the joint space to either obtain a viable synovial fluid sample or deliver drug injections [7]. Even with more experienced clinicians, joints that are less frequently operated on, such as the wrists, may still prove difficult due to lack of practice. This issue is exacerbated in specialties such as emergency medicine where clinicians tend to perform such procedures less frequently [8, 9]. As such, methods for joint access training are necessary for both procedural learning for novice clinicians as well as practice for experienced clinicians [10].

One potential technique to improve success of small joint arthrocentesis is the use of ultrasound imaging [11]. A study of joint aspiration in a 60-patient population found that rheumatologists performed successful joint aspirations in 97% of joints when using ultrasound guidance, compared to only a 32% success rate when relying solely on anatomical landmarks with no imaging [12]. The joints aspirated in this study included small joints such as the wrist and more commonly aspirated joints like shoulder, knee, and elbow joints. Specifically, ultrasound guided aspiration in small joints resulted in a 100% successful aspiration rate while aspiration of the wrist and other small joints with traditional methods was successful only 20% of the time. Furthermore, another investigation that involved residents completing a cadaveric training session found that while success rates were relatively similar between ultrasound-guided and landmark-guided arthrocentesis procedures, resident confidence in ultrasound-guided arthrocentesis was significantly higher than that of landmark-guided arthrocentesis after training [13]. Due to the high success rate and increased clinician confidence levels, it is imperative that residents and attendings master ultrasound-guided arthrocentesis for successful procedures in small joints, such as the wrist.

Thus, there is a clinical need that unpracticed clinicians performing joint aspiration and injection need to improve procedural competence in order to successfully enter the joint space and minimize damage to surrounding anatomical structures. Clinical simulation training has the potential to enable trainees to achieve the technical and non-technical competencies necessary to safely perform the arthrocentesis procedure on a real patient [14]. However, despite several joint access task trainers having been developed, there are still significant issues. For one, these devices are incredibly expensive, costing thousands of dollars each; this pricing level may be difficult to afford for teaching hospitals without explicit simulation budgets and centers. Additionally, some trainers (i.e., *Limbs & Things* elbow model) lack biofidelity, particularly as it pertains to ultrasound compatibility [15]. Existing task trainers tend to model larger and more easily accessible joints like the knee. While knee aspirations are among the most common, practice for more rare and complex joints is perhaps even more critical. Finally, a common issue seen while using existing ultrasound task trainers is the presence of needle “trails” resulting from past use. Thus, when these trainers are reused, they do not allow users to identify the optimal route for needle injection on their own. In this paper, we discuss the design and manufacture of a wrist joint access task trainer that is low-cost, ultrasound compatible, and easily reproducible with sustainable materials. This study aimed to develop a do-it-yourself (DIY) ultrasound-compatible task trainer for wrist joint access training.

## Methods

This section is separated into three parts: (1) establishing the design requirements for an effective task trainer, (2) the physical construction of the task trainer, and (3) the ultrasonic testing of the task trainer.

## Establishment of design requirements

The design and development of the device were based on a User-Centered Design Process as used in *blinded institution* [16, 17]. We were approached by emergency physicians from *blinded* hospital who were concerned about "*the lack of access to ultrasonic arthrocentesis task trainers and their poor performance*". The user-centered design process for this task trainer focused on involving the users in every stage of the process, from understanding the clinical need, to the feedback and iteration of prototypes. Here, we explain each of the stages.

### Understanding the clinical need

To understand the clinical need, we first engaged in a literature review to understand the physiopathology related to joint aspiration and literature related to simulation and procedural training in medicine. We reviewed over 50 + literature sources that could be found in the PUB MED repository in the last 20 years. With this information, we immersed ourselves in the field by using applied ethnographic methods such as open-ended interviews and participant and non-participant observation in the field [18–20]. The sample involved more than 20 clinical stakeholders including specialists in emergency medicine, rheumatology, orthopedics, hospitalist, simulation centers, and patients. We were able to triangulate frequent flaws discovered not just in the clinical technique but also in the instruction on performing the procedure. Shadowing the injection procedures and aspirations at the *blinded* Musculoskeletal Ultrasound and Injection Clinic, as well as the Emergency Department, enabled us to develop a preliminary Hierarchical Task Analysis (HTA) [21]. The HTA outlines the step-by-step goals, operations, and steps for an ideal arthrocentesis procedure (Additional file 1: Appendix S1).

### Synthesizing information to establish design requirements

After reviewing our transcribed data, the literature review, and the statement regarding our need, we converged in design requirements. These are conditions that the product needs to do to be successful and resonate with the final users [22–24]. The following Table 1 summarizes specific design requirements and the design decisions from our trainer to accomplish the requirement. Requirements are broken down into “Must Haves” and “Nice to Haves” based on how important it is to be achieved for the basic prototype.

**Table 1:** Design requirements (DR) and corresponding design aspects in our task trainer model

	Design requirements	Corresponding design aspect
Must haves	**DR01. Haptic Feedback:** The user is able to physically feel the task trainer and identify key landmarks during palpation as they are performing the procedure	The task trainer model is made of an anatomically accurate orientation of the wrist bones encased in a gelatin mixture to simulate palpable, soft tissue
	**DR02. Anatomical Accuracy Under Ultrasound (US):** The user is able to recognize anatomically relevant landmarks when guiding the needle under US imaging so that these skills can be transferred to a real-life procedure	The materials used to make our task trainer are ultrasound compatible. Clear anatomical markers to find the space in between hyperechoic bones are present and show in our images
	**DR03. Visual Feedback:** As a visible indicator of a successful procedure, the user should observe fluid taken out of the joint capsule	The colored fluid housed within our task trainer’s joint capsule may be taken out and refilled with a needle and syringe
Nice to haves	**DR04. Reusability:** The trainer allows for many needle practices without losing usability. A major problem with today's "reusable" trainers is that following needle penetration, there is a visible track where the needle traveled under ultrasonography	Our task trainer model is constructed with components that are sustainable, easily accessible, and simple to use. Each practice session, a new task trainer can be created. The wrist bones can easily be removed from gelatin and reinserted into the mold. Gelatin is then poured into this mold to create a new task trainer with no previous needle trails
	**DR05. Cost-Effective:** Task trainers may prove to be a financial burden for under-resourced and under-funded medical departments. The trainer should be cheaper than current ones on the market (< $2000)	The materials used are cheap and can be easily found online/the grocery store. Our task trainer is presented as a DIY model that does not require any special materials or manufacturing experience

Design requirements (DR) and corresponding design aspects in our task trainer model

## Construction of 3D wrist task trainer

The task trainer model consists of bones, a joint capsule, and soft tissue that emulates the flesh and skin. These were created by using resin casting, a rapid prototyping technique where a mold is designed, built, and used to pour resin that will solidify as a three-dimensional object [25].

### Task trainer mold

**Fig. 1.**
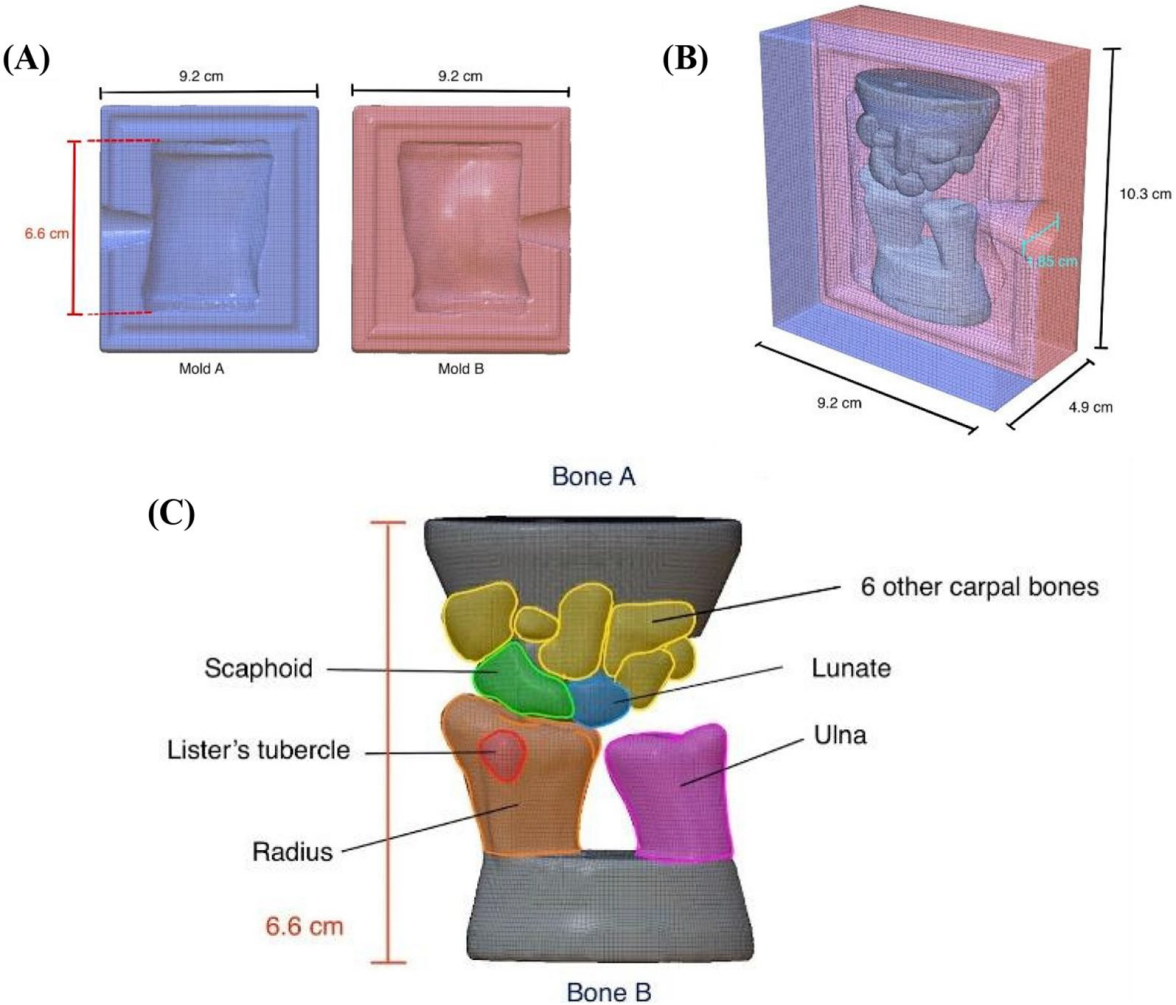
3D Design of Mold and Carpal Bones. **A** Two halves of a mold (“Mold A” and “Mold B”) were created to form an outer box that encases the two bone pieces and mimics the outer shape of the wrist. **B** The bone pieces fit within the outer mold, allowing for a watertight seal and an anatomically accurate model. Once the bones are placed within the outer mold, a polymer mixture may be poured to fill the negative space and form the soft tissue of the wrist. **C** Two conglomerated bone pieces (“Bone A” and “Bone B”) were designed and constructed following anatomical diagrams to model the many bones within the wrist. These bone pieces are placed inside the two molds prior to gelatin pouring. All 3D diagrams were created using the Nomad Sculpt platform

A mold of the wrist shape was created to allow for reproducibility of bone positioning. The mold consists of two halves and includes the negative of the two bone pieces as well as the soft tissue surrounding the wrist (Fig. 1A). Since there is only one orientation in which the bone pieces fit and allow the two halves of the mold to close, this design ensures that the bones remain stationary as the polymer material is cast. (Fig. 1B). A funnel-shaped hole measuring 1.85 cm in diameter was added to allow for easy pouring of polymer material. Easy reproducibility allows for this trainer to be remade quickly after each practice and to have a consistent appearance under ultrasound for repetitive practice.

### Modeling carpal bones

The bones are a key anatomical feature that clinicians avoid during joint access procedures, as contact with bone can cause patient discomfort. We sought to construct an anatomically accurate 3D model of the carpal bones by referencing medical diagrams from “Anatomy, Shoulder and Upper Limb, Hand Carpal Bones” [26]. From interviews and discussions with our users and key informants, it was concluded that bone protrusions (i.e., the Lister’s Tubercle) serve as anatomical landmarks during palpitation. In Fig. 1C, we see the scaphoid in green, the lunate in blue, and a singular bone mass combining six other carpal bones (“Bone A”). The distal ends of the ulna (magenta) and radius (orange) were combined into one piece to create the secondary bone mass (“Bone B”). These two larger bone masses can be placed side by side to create the joint space in the wrist where fluid withdrawal or injection occurs. To allow space to incorporate a pressure release system, channels were embedded in the two bone parts.

### Modeling joint capsule

The joint capsule is the target for injection. Under ultrasound, this capsule is best represented by a cavity in the soft tissue. Thus, we inserted an inflated balloon before casting our soft tissue polymer, which can be removed after the polymer sets to create a negative space. To satisfy the need for visual feedback, we filled the joint space with colored fluid. However, attempting to withdraw fluid from a closed fluid system results in negative pressure, making aspiration impossible. To release the pressure, we used a half-filled syringe to balance the pressure when fluid is drawn from the joints. Thus, when the user successfully enters the joint space and simultaneously pulls back on the plunger of the syringe, they will receive visual feedback from fluid entering the syringe. Our current task trainer uses food coloring and water, but incorporation of other materials to thicken the fluid may allow an even better representation of synovial fluid.

## Testing ultrasound capabilities of task trainer

To validate ultrasound compatibility and the likeliness of our task trainer under ultrasound, we used a Butterfly iQ + Ultrasound probe to capture images for analysis. The Butterfly iQ + Ultrasound probe is a portable, battery powered, ultrasound probe that can be connected to a simple application. The frequency range of this probe is 1–10 MHz. The Butterfly iQ + application was installed on an 8th generation iPad and placed on the Musculoskeletal setting. Once images were correctly positioned, they were captured and exported to FIJI for analysis. Specifically, the average pixel brightness (APB) and qualitative observations on simulated anatomical structures were analyzed.

### Modeling soft tissue

To determine the optimal composition of materials that would most closely represent human connective tissue, different ratios of materials including gelatin, agar, milk powder, castor oil, and salt were combined and assessed under ultrasound. These materials were chosen because they have been used in other ultrasound task trainers and are easily attainable and biodegradable. Thus, using these materials allows for the creation of an ultrasound compatible, replicable, and sustainable task trainer. In order to perform a quantitative assessment of each material, we developed 10 material probes and recorded the average pixel brightness (APB) and qualitative observations for each composition (Additional file 2: Appendix S2). The optimal composition was chosen based on a quantitative validation of the pixel brightness to human soft tissue and a general qualitative observation of how the material looked under ultrasound. We determined that this optimal composition consisted of 8.75% w/v gelatin, 2.5% w/v agar, and 3.5% w/v milk powder. Not only is this material visually similar to human tissue on ultrasound, but its elasticity also allows for haptic feedback because bones can be palpated through the material.

### Evaluating ultrasonic properties of task trainer

To evaluate the anatomical accuracy of the task trainer under ultrasound, images of a human wrist were captured using the Butterfly iQ + probe. Images were captured in the transverse and longitudinal planes at the center of the wrist, with the middle pointer of the Butterfly probe directly above the synovial joint capsule. The images were presented to four physicians that work in orthopedics, rheumatology, and emergency medicine to verify the identification and outline of significant anatomical structures.

The images were then imported to FIJI (version 1.54t) and converted to 8-bit images. Using the outlines identified by the experts, the average pixel brightness (APB) and area spanned for each region of interest (ROI) was identified with the ‘Measure’ tool in FIJI. Notably, the ROI denoting the synovial fluid capsule was separated into the capsule surface and synovial fluid. Captured images were imported to FIJI and significant anatomical structures were identified based on contrasts with surrounding materials as well as the relative location of the structures within the task trainer. The APB and area spanned for each ROI was then identified in the same way.

## Results

### Construction of full task trainer

**Fig. 2:**
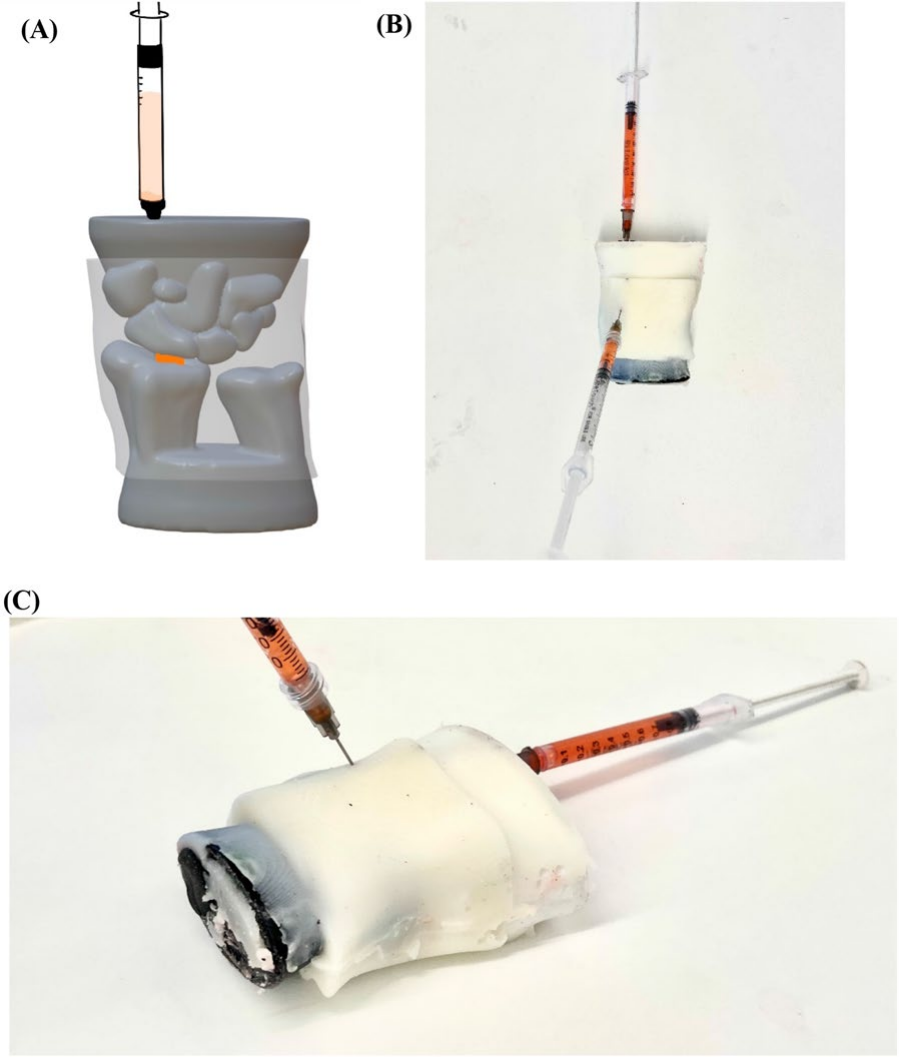
Complete Wrist Task Trainer Model. **A** Computer-generated top-down view of task trainer. The 3D bone (dark gray) pieces are held together by a casted polymer (light gray). Running through a shaft in both bone pieces is a liquid-filled balloon (orange). A liquid-filled syringe is connected to this balloon to maintain pressure. **B** Top-down view and **C** side view of a fully constructed task trainer. The secondary syringe that is puncturing the polymer in the center of the mold exemplifies a practice joint aspiration procedure being performed

Our full task trainer consists of two sets of wrist bones fully encased in a gelatin mold to simulate skin and soft tissue. Figure 2A shows a computer-generated model of a complete task trainer, while Fig. 2B and C depict a real task trainer that was fully constructed. This trainer was encased in 8.75% w/v gelatin, 2.5% w/v agar, and 3.5% w/v milk powder dissolved in water. Milk powder was added to the gelatin to increase opacity and simulate skin. A fluid-filled balloon runs through the bone and exposes a simulated synovial space in the narrow space between the bones. To relieve the pressure that builds up in the space when fluid is withdrawn, we included a syringe connected to this balloon capsule.

### Evaluation of ultrasonic properties

In comparing the average pixel brightness, the values for each corresponding anatomical structure for the task trainer made with our ideal 8.75% w/v gelatin, 2.5% w/v agar, and 3.5% w/v milk powder mixture were similar for all structures except the bones, which had an average pixel brightness of 32.5–34.5 in the human ultrasound but 75.1–90.2 in the ultrasound of the task trainer. As stated in the methods, the pixel brightness of the connective tissue in our task trainer and in the human wrist also differ, but the visual appearance is similar. The qualitative observations for the bone and joint capsule were different as well, but the regions of interest can still be identified. These observations and pixel brightness values are summarized in Table 2.

**Table 2:** Area, Pixel Brightness, and Qualitative Observations of Human Wrist and Task Trainer Under Ultrasound

Anatomical structure	Human area	Human brightness	Human qualitative observations	Task trainer area	Task trainer brightness	Task trainer Qualitative observations
Connective Tissue	89,561	137.88	Fuzzy, Hypoechoic	24,860	34.82	Slightly hazy
Scaphoid bone surface	18,304	144.326	Hyperechoic	82,001	145.717	Hyperechoic
Scaphoid sub-bone	49,308	32.524	Hypoechoic, Hazy	20,808	75.188	Hypoechoic, Hazy
Radius/ulna surface	18,768	157.421	Hyperechoic	6071	138.289	Hyperechoic
Radius/ulna sub-bone	75,252	34.581	Hypoechoic, Hazy	4170	90.228	Multiple layers of bone visible
Joint Capsule	7825	83.008	Hypoechoic, Hazy	3762	130.762	Isoechoic, Hazy (Capsule is difficult to discern)

Area, Pixel Brightness, and Qualitative Observations of Human Wrist and Task Trainer Under Ultrasound

**Fig. 3:**
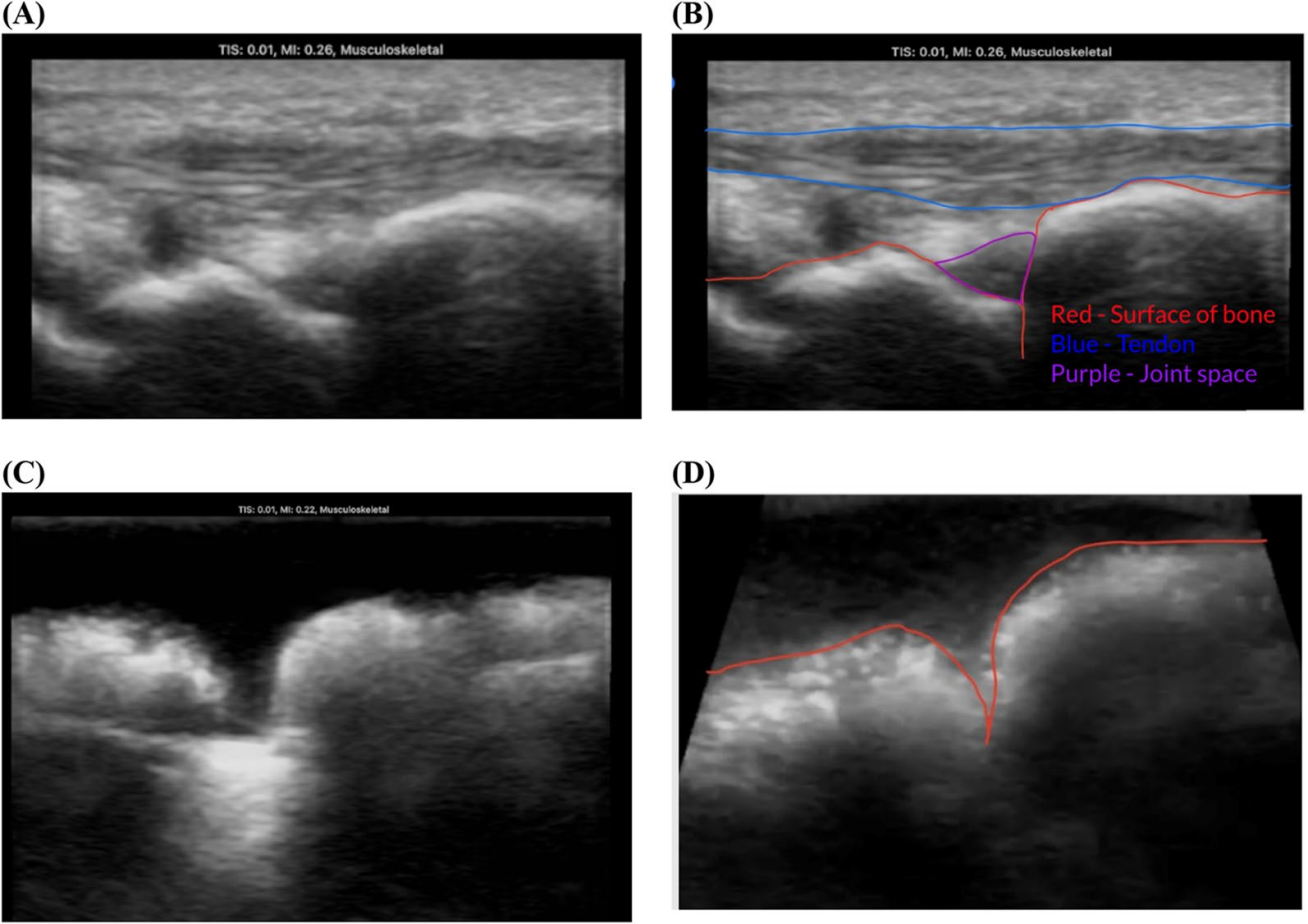
Comparison of ultrasound profiles between a human wrist and task trainer model. **A** Ultrasound profile of human wrist on longitudinal plane. Notable features in view, including the surface of the bone (red), a tendon (blue), and the joint space (purple) are outlined for reference. **B** Ultrasound profile of wrist task trainer on longitudinal plane. The surface of the 3D bone is outlined in red. Ultrasound images were captured using a Butterfly iQ + ultrasound probe on the Musculoskeletal setting

In testing the task trainer’s applicability to practicing joint aspiration procedures, we qualitatively compared the ultrasound profile of a human wrist and the corresponding ultrasound profile for our task trainer. Figure 3A and B depicts the distinctions in bone and soft tissue to locate the central joint space. This was guided by experienced physicians who assisted in identifying important features for ultrasound-guided joint aspiration. Figure 3C and D depicts the image produced when the ultrasound probe is placed on our wrist task trainer in the longitudinal plane. The bone surface of the task trainer, consisting of the radius and the ulna, was identifiable and showed similarity to the bone surface of a human wrist in terms of shape and size. However, the brightness of the simulated bone is much greater under ultrasound. In terms of differences, our task trainer did not consist of differing layers of soft tissue, such as the tendon. The appearance of the simulated soft tissue somewhat mimicked the brightness and texture of that of real human tissue. The location of the joint capsule was not immediately identifiable under ultrasound but could be estimated based on its proximity to the intersection between the radius and ulna bone pieces.

## Discussion

The Design Process allowed us to successfully develop an ultrasound compatible task trainer for wrist joint access training utilizing low-cost materials including gelatin, milk powder, and agar. When imaged under the ultrasound: bones display a well-defined white outline with a dark shadow beneath it, fluid pockets are depicted by well-defined black patches, and soft tissue are represented by striated white streaks. From the pixel brightness analysis conducted on the components of the task trainer compared to the human counterparts, we concluded that our current choice of materials can replicate the ultrasound profile of the synovial joint space and basic soft tissue. Furthermore, we were able to demonstrate the contrast between tissue types and anatomical structures when imaged under ultrasound.

Studies of task trainer models have produced similar results. Findings from the testing of a low-cost, reusable, ballistic gelatin ultrasound phantom for simulation of glenohumeral intra-articular injections concluded that ballistic gelatin showed promise for use in high-fidelity ultrasound simulations, but the task trainer still needed improvements in simulating soft tissue structures like fat, muscle, and tendon [27]. 3D-printed ultrasound phantoms have also been explored, such as a study on a tailored thyroid gland phantom for fine-needle aspiration cytology that concluded that finding materials that would best enable the manipulation of ultrasound parameters like ultrasonographic scattering and attenuation remains a challenge [28]. A different study constructed task trainers consisting of ballistic gelatin body walls that were successfully used to simulate a number of ultrasound-guided invasive procedures [29]. These studies show that existing task trainers that were constructed with similar techniques and serve similar purposes compared to our joint access training task trainer fundamentally can assist with the training of a variety of ultrasound-guided medical procedures; however, further research on better ultrasound-compatible materials and modeling of complex anatomical structures lie in the future.

Our proposed design balances the low-cost and convenient nature of a DIY task trainer and one with high fidelity and specialized features. However, we have identified some limitations with our task trainer. Particularly with the simulation of physiological landmarks in ultrasound-guided joint access, different layers of soft tissue like the tendon could be made more distinct and better match the brightness levels of corresponding structures in the human body. With regards to ultrasound visibility, we recognize that a shortcoming of conventional models is the inability to recreate the challenge of visualizing the needle under ultrasound—often one of the more difficult parts of the procedure. As our study was specifically focused on topics related to accessibility, cost, and the removal of needle tracks, we prioritized optimizing the materials in this paper with additional applications testing in the future. Additionally, the materials utilized impact lifespan and storage considerations of the task trainer. In our experience with constructing dozens of task trainers, we found that characteristics including structural integrity, physical appearance, and ultrasound visibility were impacted by time and environmental conditions, ultimately rendering the trainer unusable. When left for a long duration, and especially at room temperature, gelatin is subject to microbial growth throughout the entire task trainer. This obscures the physical view of the trainer, and is unsanitary and can lead to contamination. Thus, we opted to store finished gelatin-based trainers in a 4 °C refrigerator to delay this growth, in which we discovered that our trainers lasted up to 7 days before dehydrating to the extent that the gelatin would crack and lose its structural integrity. These characteristics were variable based on how well the gelatin mixture was incorporated and poured into the mold. Based on these findings, we recommend that fully constructed trainers awaiting use be stored in an enclosed container with a moisture source (e.g., a damp towel or a thin level of liquid) inside a 4 °C cooling unit for up to one week before disposal and recycling of plastic parts. Additionally, if the user notices any cracking or mold growth on the gelatin prior to the 7 day time point, we recommend disposal of the gelatin and recreation of a new trainer.

Through an established design and development process, we targeted the lack of resources put into the effective training of medical professionals in specialized procedures, an overlooked issue due to the often-lower frequency of such procedures and additional expenses required that strain department budgets. Furthermore, existing task trainers do not accurately replicate physiology that is important for achieving procedural competence. In recent decades, ultrasound guidance is increasingly being employed as a visualization tool for procedures involving small and/or complex structures in the body. With this ultrasound compatible task trainer model, we demonstrate a cheap, efficient method of practicing joint aspiration and injections that accurately portrays fundamental landmarks in the said procedures. The trainer is built from sustainable and easily accessible materials that enable a DIY approach for construction and practice with the device in both clinical and home settings. Since the specific anatomical structures and mold are 3D-printed and can be easily adapted, the methodologies presented in this paper may be transferable to other procedures calling for additional training of novice clinicians as well as the addition of more landmarks to be used for practice by experienced clinicians. Future work should focus on verifying how this task trainer model works in a training scenario and whether clinicians achieve the competencies required to perform this procedure on a real patient.

### Supplementary Information

**Additional file 1.** **Hierarchial Task Analysis of a Wrist Arthrocentesis Procedure.** This is a step-by-step breakdown of the necessary steps taken for a successful wrist arthrocentesis procedure and was critical for determining and validating the clinical need for joint access training.

**Additional file 2.** **Comparison of Materials for Soft Tissue.** Various combinations of gelatin, agar agar, and milk powder were imaged under ultrasound. Data was collected on pixel area, pixel brightness, and general qualitative observations of the ultrasound images.

## Data Availability

All the datasets supporting the conclusions of this article can be found directly within the manuscript and its accompanying Figures and Appendix.
