# Supplementary material for: A do it yourself (DIY) point-of-care wrist ultrasound phantom for joint access training
Source: Ultrasound J. 2024 Jun 14;16:32. doi: 10.1186/s13089-024-00374-5 (PMC11178702; doi:10.1186/s13089-024-00374-5)
Supplement: Supplementary file 1 — Additional file 1. Hierarchial Task Analysis of a Wrist Arthrocentesis Procedure. This is a step-by-step breakdown of the necessary steps taken for a successful wrist arthrocentesis procedure and was critical for determining and validating the clinical need for joint access training. [file 13089_2024_374_MOESM1_ESM.docx]

| **Successfully perform arthrocentesis of the wrist** | | | |
| --- | --- | --- | --- |
| **1: Collect supplies (score: 0)**   - - Ultrasound   - Chlorhexidine   - Lidocaine 1%   - 5cc syringe w/ 25-gauge needle   - 5cc syringe w/ 18–20-gauge needle   - Specimen cup   - Probe cover   - Sterile gel packet   - Sterile gloves   - Gauze/ tape | | | |
| **2: Inspect joint** | | | |
|  | 2.1 Position the wrist appropriately | | |
|  |  | 2.11 Hand pronated, | |
|  |  | 2.12 Hand slightly flexed (20-50^o^) | |
|  |  | 2.13 Hand ulnar deviation | |
|  |  | 2.14 Roll under palmar wrist to allow hand to naturally fall into this position | |
|  | 2.2 Identify anatomic landmarks | | |
|  |  | 2.21 Identify Lister’s tubercle | |
|  |  | 2.22 Identify extensor pollicis longus (EPL) tendon (ulnar border of anatomic snuffbox) | |
|  |  | 2.23 Identify entry site distal to Lister’s tubercle and lateral to EPL tendon | |
|  | 2.3 Identify needle insertion site using ultrasound | | |
|  |  | 2.31 Select high frequency linear probe in MSK exam mode | |
|  |  | 2.32 Identify on ultrasound the following structures: | |
|  |  |  | 2.321 radius |
|  |  |  | 2.322 adjacent carpal bone |
|  |  |  | 2.323 synovial fluid |
|  |  | 2.33 Identify largest pocket of radiocarpal joint fluid | |
|  |  | 2.35 Select and mark optimal site | |
| **3: Sterile preparation** |  |  | |
|  | 3.1 Apply chlorhexidine in progressively larger concentric circles around the marked site | | |
|  | 3.2 Don sterile gloves | | |
|  | 3.3 Place sterile drape/towels around the marked site | | |
|  | 3.4 Place a sterile probe cover on ultrasound transducer | | |
| **4: Anesthetize the needle puncture site** |  | | |
|  | 4.1 Draw 5ml of lidocaine into a syringe and attach 25-gauge needle | | |
|  | 4.2 Create skin wheal | | |
|  | 4.3 Inject lidocaine at the marked spot along the anticipated track of the aspiration needle | | |
|  |  | | |

| **5: Enter radiocarpal joint space** | | |
| --- | --- | --- |
|  | 5.1 Re-identify sonographic landmarks in longitudinal axis of wrist joint (radius, carpal bone, fluid pocket, and depth to fluid pocket) | |
|  | 5.2 Select 18-20-gauge needle attached to syringe | |
|  | 5.2 Enter radiocarpal joint space | |
|  |  | 5.21 Puncture skin at a steep (70-90^o^) angle using an out-of-plane approach (Alternative: insert needle using an in-plane approach at a shallower (45^o^) angle where the distal radius acts as a bony backstop. If in-plane approach is used, the probe may need to be slightly rotated to best target fluid collection.) |
|  |  | 5.22 Visualize needle tip as it enters the joint effusion |
|  |  | 5.23 Aspirate as needle advances through soft tissue |
|  | 5.3 Aspirate joint fluid | |
|  |  | 5.32 Aspirate as much fluid as possible |
|  |  | 5.33 Perform troubleshooting if fluid stops flowing, e.g.   - - - Slightly advance or retract needle     - Rotate the bevel     - Lessen force of aspiration     - Provide traction to the joint     - Milk the effusion |
| **6: Send specimen for appropriate testing** | | |
|  | 6.1 Place synovial fluid in sterile specimen cup | |
|  | 6.2 Order appropriate testing for clinical indication | |
