# Supplementary material for: A do it yourself (DIY) point-of-care wrist ultrasound phantom for joint access training
Source: Ultrasound J. 2024 Jun 14;16:32. doi: 10.1186/s13089-024-00374-5 (PMC11178702; doi:10.1186/s13089-024-00374-5)
Supplement: Supplementary file 2 — Additional file 2. Comparison of Materials for Soft Tissue. Various combinations of gelatin, agar agar, and milk powder were imaged under ultrasound. Data was collected on pixel area, pixel brightness, and general qualitative observations of the ultrasound images. [file 13089_2024_374_MOESM2_ESM.docx]

**Appendix 2. Comparison of Materials for Soft Tissue.**

Various combinations of gelatin, agar agar, and milk powder were imaged under ultrasound. Data was collected on pixel area, pixel brightness, and general qualitative observations of the ultrasound images. The highlighted combination indicates the option that we selected for our soft tissue composition.

| **Material Combination** | **Area** | **Pixel Brightness** | **Qualitative Observations** |
| --- | --- | --- | --- |
| 5x Gelatin | 105513 | 2.052 | Extremely dark with corners refracting US waves |
| 5x Gelatin with 17.5g Milk Powder | 117961 | 62.346 | Grey, hazy, unstructured, random bright patches |
| 5x Gelatin with 7g Milk Powder | 109510 | 38.309 | Grey, hazy, unstructured, random bright patches |
| 5x Gelatin with 3.5g Milk Powder | 89580 | 30.509 | Grey, hazy, unstructured |
| 5x Gelatin with 1.75g Milk Powder | 90264 | 11.484 | Slightly grey, mostly dark |
| 5x Gelatin with 1g Agar and 3.5g Milk Powder | 35456 | 76.03 | Grey, hazy, unstructured, top half of block is not the same as bottom half (material settled) |
| 5x Gelatin with 2.5g Agar and 2.5g Milk Powder | 35961 | 50.142 | Grey, hazy, unstructured, top half of block is not the same as bottom half (material settled) |
| 5x Gelatin with 2.5g Agar and 3.5g Milk Powder | 73106 | 53.387 | Grey, hazy, unstructured, not many bright patches, does not have issue with top half of block not showing up |
| 5x Gelatin with 5g Agar | 128445 | 104.894 | Grey, hazy, unstructured, a few bright patches, has an observable gradient of brightness |
| 5x Gelatin with 7.5g Agar | 112445 | 117.457 | Grey, hazy, unstructured, a few bright patches, has an observable gradient of brightness |
